# Supplementary material for: Effect of Rowachol on the Gallbladder Dysmotility Disorder Based on Gallbladder Ejection Fraction
Source: Medicina (Kaunas). 2023 Jan 3;59(1):105. doi: 10.3390/medicina59010105 (PMC9862614; doi:10.3390/medicina59010105)
Supplement: Supplementary file 1 [file medicina-59-00105-s001.zip › medicina-2111246-supplementary.pdf]

Supplementary Table S1. Co-medication list during Rowachol® treatment.

Oral bile acids [1]	24 (77.4%)
Proton pump inhibitor/Potassium competitive acid blocker [2]	20 (64.5%)
Motilitone Tab.® (Corydalis Tuber & Pharbitidis Semen)	18 (58.1%)
Octylonium bromide/Pinaverium bromide	14 (45.2%)
Itopride hydrochloride	8 (25.8%)
Probiotics [3]	6 (19.4%)
Stillen Tab.® (Artemisia Herb 95% Ethanol Soft Ext)	2 (6.5%)
Camostat mesilate	2 (6.5%)
Alprazolam	1 (3.2%)

[1]: Includes Ursa ® (ursodeoxycholic acid) and CNU ® (magnesium trihydrate salt of chenodeoxycholic acid and ursodeoxycholic acid)

[2]: Includes Omeprazole, Ilapazole, Esomeprazole and Tegoprazan

[3]: Includes Lacidofil Cap.® (bacteria culture of Lactobacillus rhamnosus R0011 and Lactobacillus helveticus R0052 20 mg) and Medilac-DS Enteric Coated Cap.® (bacillus subtilis·streptococcus faecium)
